# Similarities, reliability and gaps in assessing the quality of conduct of systematic reviews using AMSTAR-2 and ROBIS: systematic survey of nutrition reviews

**DOI:** 10.1186/s12874-021-01457-w

**Published:** 2021-11-27

**Authors:** Mateusz J. Swierz, Dawid Storman, Joanna Zajac, Magdalena Koperny, Paulina Weglarz, Wojciech Staskiewicz, Magdalena Gorecka, Anna Skuza, Adam Wach, Klaudia Kaluzinska, Justyna Bochenek-Cibor, Bradley C. Johnston, Malgorzata M. Bala

**Affiliations:** 1 Chair of Epidemiology and Preventive Medicine, Department of Hygiene and Dietetics, Jagiellonian University Medical College, Kopernika 7 Street 31-034, Krakow, Poland; 2 Chair of Epidemiology and Preventive Medicine, Department of Epidemiology Jagiellonian University Medical College , Kopernika 7 Street 31-034, Krakow, Poland; 3 Students’ Scientific Research Group of Systematic Reviews, Jagiellonian University Medical College, Krakow, Poland; 4 Department of Radiation Oncology, St Lukas Hospital, Tarnow, Poland; 5 Departments of Nutrition, Epidemiology and Biostatistics, Texas A&M University, College Station, College Station, TX USA; 6 Department of Health Research Methods, Evidence, and Impact, McMaster University, Hamilton, Ontario Canada

**Keywords:** AMSTAR-2, ROBIS, Quality, Systematic review, Meta-analysis, Nutrition, Cancer prevention

## Abstract

**Background:**

AMSTAR-2 (‘A Measurement Tool to Assess Systematic Reviews, version 2’) and ROBIS (‘Risk of Bias in Systematic Reviews’) are independent instruments used to assess the quality of conduct of systematic reviews/meta-analyses (SR/MAs). The degree of overlap in methodological constructs together with the reliability and any methodological gaps have not been systematically assessed and summarized in the field of nutrition.

**Methods:**

We performed a systematic survey of MEDLINE, EMBASE, and the Cochrane Library for SR/MAs published between January 2010 and November 2018 that examined the effects of any nutritional intervention/exposure for cancer prevention. We followed a systematic review approach including two independent reviewers at each step of the process. For AMSTAR-2 (16 items) and ROBIS (21 items), we assessed the similarities, the inter-rater reliability (IRR) and any methodological limitations of the instruments. Our protocol for the survey was registered in PROSPERO (CRD42019121116).

**Results:**

We found 4 similar domain constructs based on 11 comparisons from a total of 12 AMSTAR-2 and 14 ROBIS items. Ten comparisons were considered fully overlapping. Based on Gwet’s agreement coefficients, six comparisons provided almost perfect (> 0.8), three substantial (> 0.6), and one a moderate level of agreement (> 0.4). While there is considerable overlap in constructs, AMSTAR-2 uniquely addresses explaining the selection of study designs for inclusion, reporting on excluded studies with justification, sources of funding of primary studies, and reviewers’ conflict of interest. By contrast, ROBIS uniquely addresses appropriateness and restrictions within eligibility criteria, reducing risk of error in risk of bias (RoB) assessments, completeness of data extracted for analyses, the inclusion of all necessary studies for analyses, and adherence to predefined analysis plan.

**Conclusions:**

Among the questions on AMSTAR-2 and ROBIS, 70.3% (26/37 items) address the same or similar methodological constructs. While the IRR of these constructs was moderate to perfect, there are unique methodological constructs that each instrument independently addresses. Notably, both instruments do not address the reporting of absolute estimates of effect or the overall certainty of the evidence, items that are crucial for users’ wishing to interpret the importance of SR/MA results.

**Supplementary Information:**

The online version contains supplementary material available at 10.1186/s12874-021-01457-w.

## Background

With the ever-growing amount of published data, systematic reviews (SRs) and meta-analyses (MAs) became recognised methods for summarising the evidence in support of evidence-based decision-making in healthcare [1–3]. High quality systematic reviews/meta-analyses (SR/MAs) are considered acceptable and important for decision-makers [4, 5]. However, with the increasing number of SR/MAs there are often issues of reliability, particularly when SR/MAs have conflicting results and suffer from extensive methodological shortcomings [1, 6, 7]. In the context of these findings, users of the literature must distinguish lower versus higher quality SR/MAs to support healthcare decision-making. Instruments to distinguish the quality of conduct of SR/MAs have been designed and validated.

Currently, two instruments, namely AMSTAR-2 (‘A Measurement Tool to Assess Systematic Reviews, version 2’) and ROBIS (‘Risk of Bias in Systematic Reviews’), are commonly used to formally assess the quality of conduct of SR/MAs. Both instruments provide a structured approach for readers to perform rapid and reproducible assessments of the quality, including a detailed evaluation of conduct and methodological rigour; however original constructs and specific details differ [8, 9]. AMSTAR-2 has been developed as a critical appraisal tool for SR/MAs that include randomised or non-randomised studies of health care interventions and is an updated version of previously widely accepted AMSTAR that has been in use for over a decade [10]. AMSTAR-2 is comprised of 16 items, of which seven were determined to be critically important to the validity of a review, while the other nine are considered not critically important. Users of AMSTAR-2 are asked to make an overall judgment of ‘high’, ‘moderate’, ‘low’, or ‘very low’ confidence in the results of SR/MA based on the assessment of critical and non-critical items [11].

ROBIS focuses intrinsically on the risk of bias (RoB) in the SR/MA and comprises three phases: assessment of relevance (optional), identification of concerns within the review process that put the SR/MA at RoB, and judgement of RoB. The second of the aforementioned phases is composed of four domains with 21 items highlighting specific issues that need to be considered. In the third phase a judgement of ‘low’, ‘high’, or ‘unclear’ RoB is assigned after consideration of assessments performed in the second phase [12].

Upon applying both instruments, users can determine that they are similar in their general approach; however, differences do exist. A number of studies have investigated the similarity of assessments between original AMSTAR and ROBIS tools [13–15]. Nevertheless, so far, only one study has investigated the comparability of both instruments in terms of their domains and corresponding items, demonstrating a satisfactory correlation between the overall ratings of AMSTAR-2 and ROBIS while highlighting the differences in the conceptual frameworks of both tools [16].

There has been a profusion of SR/MAs in the health sciences literature [1], with several studies having already investigated their quality [7, 17, 18]. Nutritional epidemiology is an area of scientific interest to the public, and while the quality of SR/MAs in the field has recently been shown to be sub-optimal [7], the related and burgeoning field of SR/MAs assessing nutrition for cancer prevention has not been systematically evaluated. In this study, performed within the context of the systematic survey addressing trustworthiness of SR/MAs assessing nutrition for cancer prevention, we aimed to compare the similarities, the inter-rater reliability (IRR) and any methodological gaps of instruments for assessing the quality of conduct of those SR/MAs.

## Methods

The protocol for the systematic survey was prepared a priori and registered in PROSPERO with an identification number CRD42019121116.

### Searches, eligibility, and sample selection

We systematically searched MEDLINE, Embase, and the Cochrane Library for SR/MAs published between January 2010 and November 2018 that examined the effects of any nutritional intervention/exposure for cancer prevention in the general population or in people at higher risk for cancer. Search strategies are provided in Supplementary file. We accepted studies labelled as SR/MAs as described in the title, abstract, or full text, which included, according to their eligibility criteria, primary studies comprising a comparator group (i.e., interventional studies with a control group such as randomised or non-randomised controlled trials, observational studies with participants categorized by intake or exposure level (e.g. lower versus upper quartiles)). The methods have been described in detail in the companion paper [19].

### Screening and data extraction

Following a calibration exercise, pairs of two independent reviewers performed study selection, data extraction, as well as both AMSTAR-2 and ROBIS assessments, with conflicts resolved by discussion or consultation with a third reviewer. Each step was preceded with calibration exercises to ensure common understanding of inclusion criteria and to discuss any ambiguities. With respect to quality assessments, a number of authors have considerable experience in conducting SRs and assessing their methodological quality (MJS, DS, JZ, MK, BCJ, MMB), while the remaining authors (PT, WS, MG, AS, AW, KK, JBC) underwent training. AMSTAR-2 and ROBIS assessments were piloted on a set of three studies.

### Quality of conduct and risk of bias assessment instruments

AMSTAR-2 consists of 16 items for which ‘yes (Y)’ or ‘no (N)’ judgments can be applied. For five items (2, 4, 7, 8, 9) in addition to ‘Y’ or ‘N’ responses, ‘partially yes (PY)’ can be selected. Items 11, 12, and 15 are not considered if a meta-analysis was not undertaken. Among the 16 items, seven are considered to be critical: ‘development of the study protocol’ (item 2); ‘comprehensiveness of the literature search strategy’ (item 4); ‘providing a list of excluded studies with reasons’ (item 7); ‘appropriate assessment of the RoB of individual included studies’ (item 9); ‘use of appropriate meta-analytical methods’ (item 11); ‘consideration of RoB when interpreting and discussing the results’ (item 13); and ‘assessment of the presence of publication bias and discussion of its impact on the results’ (item 15). The remaining nine items are considered non-critical. Subsequent to judging the 16 items, investigators can make an overall judgment of ‘high’, ‘moderate’, ‘low’, or ‘very low’ confidence in the results of the target SR/MA, as follows [11]:

- *High*: no major flaws in critical items and ≤ 1 flaw in non-critical items;
- *Moderate*: no major flaws in critical items and > 1 flaw in non-critical items;
- *Low*: one major flaw in critical items with or without non-critical items;
- *Critically low*: > 1 major flaw in critical items with or without non-critical items.

ROBIS consists of 21 items assigned to four domains (study eligibility criteria; identification and selection of studies; data collection and study appraisal; synthesis and findings), for which respondents can answer ‘yes (Y)’, ‘partial yes (PY)’, ‘partial no (PN)’, ‘no (N)’, or ‘no information (NI)’. The overall concerns associated with each of the four domains are then judged as ‘low’, ‘high’ or ‘unclear’. On the basis of the domain assessments, supported by consideration of correctness of SR/MA interpretation of findings, relevance of included studies to the SR/MA’ question, as well as fairness and thoroughness within presentation of the results, a final consideration is performed on whether the SR/MA as a whole is at ‘low’, ‘high’, or ‘unclear’ risk of bias [12].

### Domain matching

For data collection and analyses we used Microsoft Excel (version 2016). After reviewing all items of each instrument, based on the ROBIS instrument we categorized the items under four main domains based on conceptual similarities:

- Domain 1: Study eligibility criteria;
- Domain 2: Identification and selection of studies;
- Domain 3: Data collection and study appraisal;
- Domain 4: Synthesis and findings.

After assessing the concept, approach and definitions for each item, we matched items from each instrument to produce 11 comparisons including 12 AMSTAR-2 and 14 ROBIS items. In some cases, two or more items from one of the instruments were combined within a single comparison (e.g. AMSTAR-2 item 4 was compared with ROBIS items 2.1, 2.2, 2.3, and 2.4). For 10 comparisons we judged items of both instruments as satisfactorily comparable with respect to concept, approach and definitions, while in the case of one comparison (examination of publication bias/robustness of the results) we judged the items from the instruments as only partially overlapping (i.e. robustness of SR/MA results includes an assessment of publication bias as well as other considerations). There were four items on AMSTAR-2 and seven items on ROBIS that did not sufficiently overlap in concept, approach, and description. Table 1 provides a summary of the overlapping and non-overlapping items.

**Table 1:** Comparison of matched AMSTAR-2 and ROBIS items

AMSTAR-2	ROBIS	Level of agreement; Gwet’s AC1 (95% CI)
**Domain 1: Study eligibility criteria**		
1. Did the research questions and inclusion criteria for the review include the components of PICO?	1.3 Were eligibility criteria unambiguous?	Almost perfect; 0.87 (0.78, 0.96)
2. Did the report of the review contain an explicit statement that the review methods were established before the conduct of the review and did the report justify any significant deviations from the protocol?	1.1 Did the review adhere to predefined objectives and eligibility criteria? (protocol)	Almost perfect; 0.99 (0.97, 1)^a^
3. Did the review authors explain their selection of the study designs for inclusion in the review?	Not considered	–
Not considered	1.2 Were the eligibility criteria appropriate for the review question?	–
Not considered	1.4 Were all restrictions in eligibility criteria based on study characteristics appropriate?	–
Not considered	1.5 Were any restrictions in eligibility criteria based on sources of information appropriate?	–
**Domain 2: Identification and selection of studies**		
4. Did the review authors use a comprehensive literature search strategy?	2.1 Did the search include an appropriate range of databases/electronic sources for published and unpublished reports? 2.2 Were methods additional to database searching used to identify relevant reports? 2.3 Were the terms and structure of the search strategy likely to retrieve as many eligible studies as possible? 2.4 Were restrictions based on date, publication format, or language appropriate?	Substantial; 0.79 (0.74, 0.85)*^b^
5. Did the review authors perform study selection in duplicate?	2.5 Were efforts made to minimize error in selection of studies?	Almost perfect; 0.87 (0.77, 0.96)
Not considered	3.5 Were efforts made to minimize error in risk of bias assessment?	–
**Domain 3: Data collection and study appraisal**		
6. Did the review authors perform data extraction in duplicate?	3.1 Were efforts made to minimize error in data collection?	Almost perfect; 0.88 (0.79, 0.98)
7. Did the review authors provide a list of excluded studies and justify the exclusions?	Not considered	–
8. Did the review authors describe the included studies in adequate detail?	3.2 Were sufficient study characteristics available for both review authors and readers to be able to interpret the results?	Moderate; 0.6 (0.44, 0.76)^a^
Not considered	3.3 Were all relevant study results collected for use in the synthesis?	–
9. Did the review authors use a satisfactory technique for assessing the risk of bias (RoB) in individual studies that were included in the review?	3.4 Was risk of bias (or methodological quality) formally assessed using appropriate criteria?	Almost perfect; 0.88 (0.79, 0.98)^c^
10. Did the review authors report on the sources of funding for the studies included in the review?	Not considered	–
**Domain 4: Synthesis and findings**		
Not considered	4.1 Did the synthesis include all studies that it should?	–
Not considered	4.2 Were all predefined analyses reported or departures explained?	–
11. If meta-analysis was performed, did the review authors use appropriate methods for statistical combination of results?	4.3 Was the synthesis appropriate, given the nature and similarity in the research questions, study designs, and outcomes across included studies? 4.4 Was between-study variation (heterogeneity) minimal or addressed in the synthesis?^d^	Almost perfect; 0.81 (0.69, 0.92)^e^
12. If meta-analysis was performed, did the review authors assess the potential impact of RoB in individual studies on the results of the meta-analysis or other evidence synthesis? 13. Did the review authors account for RoB in individual studies when interpreting/discussing the results of the review?	4.6 Were biases in primary studies minimal or addressed in the synthesis?	Substantial; 0.77 (0.64, 0.89)^f^
14. Did the review authors provide a satisfactory explanation for, and discussion of, any heterogeneity observed in the results of the review?	4.4 Was between-study variation (heterogeneity) minimal or addressed in the synthesis?^d^	Substantial; 0.73 (0.59, 0.86)
15. If they performed quantitative synthesis, did the review authors carry out an adequate investigation of publication bias (small study bias) and discuss its likely impact on the results of the review?	4.5 Were the findings robust, for example, as demonstrated through funnel plot or sensitivity analyses?	Slight; 0.18 (−0.03, 0.38)^g,h^
16. Did the review authors report any potential sources of conflict of interest, including any funding they received for conducting the review?	Not considered	–

95% CI - 95% confidence interval

Gwet’s AC1 - Gwet’s first-order agreement coefficient

ROBIS ‘Y’ and ‘PY’ corresponded to AMSTAR-2 ‘Y’, while ROBIS ‘N’, ‘PN’, and ‘NI’ corresponded to AMSTAR-2 ‘N’

(a) ‘Y’ and ‘PY’ in AMSTAR-2 equalled ‘Y’ and ‘PY’ in ROBIS. ‘N’ in AMSTAR-2 equalled ‘N’, ‘PN’, and ‘NI’ in ROBIS

(b) ‘PY’ in AMSTAR-2 equalled ‘Y’ and ‘PY’ in ROBIS provided these were assessed accordingly both in ROBIS items 2.3 and 2.4. ‘Y’ in AMSTAR-2 equalled ‘Y’ and ‘PY’ in ROBIS provided these were assessed accordingly in ROBIS items 2.1, 2.2, 2.3, and 2.4. Otherwise, ROBIS judgements equalled ‘N’ in AMSTAR-2

(c) If the study included randomized controlled studies (RCTs) only or non-randomised studies of interventions/exposures (NRSI) only: ‘Y’ and ‘PY’ in AMSTAR-2 equalled ‘Y’ and ‘PY’ in ROBIS; ‘N’ in AMSTAR-2 equalled ‘N’, ‘PN’, and ‘NI’ in ROBIS. If the study included both RCTs and NRSI: ‘Y’ or ‘PY’ for both RCTs and NRSI in AMSTAR-2 equalled ‘Y’ and ‘PY’ in ROBIS. If RCTs and/or NRSI scored ‘N’ in AMSTAR-2 it equalled ‘N’, ‘PN’, and ‘NI’ in ROBIS

(d) ROBIS item 4.4 was used for two comparisons

(e) Not considered if no MA in the study. If there was MA including RCTs only or NRSI only: ‘Y’ in AMSTAR-2 equalled ‘Y’ and ‘PY’ in ROBIS. ‘N’ in AMSTAR-2 equalled ‘N’, ‘PN’, and ‘NI’ in ROBIS. If the study included MA of RCTs and NRSI: ‘Y’ for both RCTs and NRSI in AMSTAR-2 equalled ‘Y’ and ‘PY’ in ROBIS. If RCTs and/or NRSI scored ‘N’ in AMSTAR-2 it equalled ‘N’, ‘PN’, and ‘NI’ in ROBIS

(f) If there was no MA only AMSTAR-2 item 13 was compared with ROBIS item 4.6: ‘Y’ in AMSTAR-2 equalled ‘Y’ and ‘PY’ in ROBIS. ‘N’ in AMSTAR-2 equalled ‘N’, ‘PN’, and ‘NI’ in ROBIS. If the study included MA: ‘Y’ in both AMSTAR-2 item 12 and 13, this equalled ‘Y’ and ‘PY’ in ROBIS. If AMSTAR-2 item 12 or 13 scored ‘N’, this equalled ‘N’, ‘PN’, and ‘NI’ in ROBIS

(g) Not considered if no MA in the study

(h) This comparison was considered partially overlapping

* quadratic weights

### Reliability

For comparing the similar items across instruments, using the Gwet’s AC1 statistic (Gwet’s first-order agreement coefficient) we calculated the reliability between raters [20, 21]. In order to do so, pairs of reviewers independently assessed each SR/MA using AMSTAR-2 and ROBIS. When we found ambiguities in our assessments, we discussed, and if we could not come to consensus a third senior reviewer was consulted. Subsequently, items with consensus appraisals for each study were used to calculate the IRR. Assumptions for each comparison are provided in the footnotes of the Table. 1. Based on established guidance, we classified agreement as poor (≤ 0.00), slight (0.01–0.20), fair (0.21–0.40), moderate (0.41–0.60), substantial (0.61–0.80), and almost perfect (0.81–1.00) [16, 22].

## Results

**Fig. 1:**
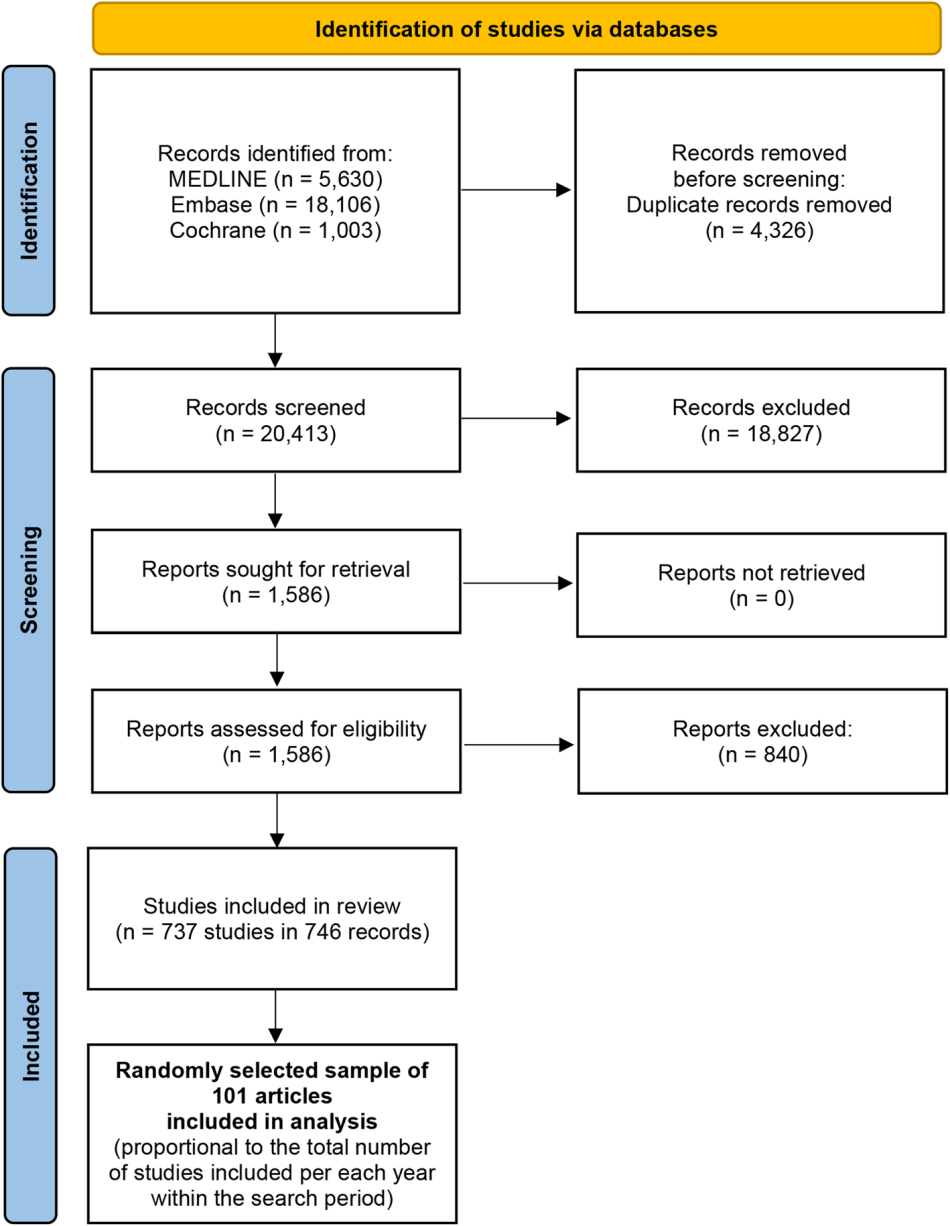
PRISMA 2020 flow diagram. PRISMA - Preferred Reporting Items for Systematic Reviews and Meta-Analyses

We identified 24,739 records, of which 20,413 were screened after duplicates were removed. Based on the eligibility criteria, we included 737 studies, of which a random sample of 101 articles was selected and analysed. The study flow is presented in Fig. 1 [23].

The 11 comparisons produced varying levels of agreement coefficients, presented below.

### Domain 1: study eligibility criteria

Two comparisons were created within this domain. The comparisons addressed the comprehensiveness of eligibility criteria and the prospective publication of review methods (protocol), with an almost perfect agreement: 0.87 (95% CI, 0.78 to 0.96) and 0.99 (95% CI, 0.97 to 1), respectively.

### Domain 2: identification and selection of studies

Two comparisons were discerned within this domain. One addressed the comprehensiveness of search strategies with a substantial level of agreement: 0.79 (95% CI, 0.74 to 0.85), and the other investigated duplicate study selection with an almost perfect level of agreement: 0.87 (95% CI, 0.77 to 0.96).

### Domain 3: data collection and study appraisal

Three comparisons were formed within this domain. One addressed duplicate data extraction with an almost perfect level of agreement: 0.88 (95% CI, 0.79 to 0.98). A second comparison explored the comparability of items regarding the adequate description of characteristics of studies included in the review showing a moderate level of agreement: 0.6 (95% CI, 0.44 to 0.76). A third comparison addressed the use of appropriate RoB assessment methods showing an almost perfect level of agreement: 0.88 (95% CI, 0.79 to 0.98).

### Domain 4: synthesis and findings

Four comparisons were created within this domain. Three were considered fully overlapping, while one was partially overlapping. One comparison, concerning an appropriate statistical combination of results, proved an almost perfect level of agreement: 0.81 (95% CI, 0.69 to 0.92). Two comparisons, one regarding assessment and interpretation of biases in included studies, and one concerning appropriate consideration of heterogeneity within the results, both showed substantial levels of agreement: 0.77 (95% CI, 0.64 to 0.89) and 0.73 (95% CI, 0.59 to 0.86), respectively. The fourth comparison addressing publication bias and robustness of the results (e.g. funnel plot or sensitivity analysed) was considered as partially overlapping and showed a slight level of agreement: 0.18 (95% CI, − 0.03 to 0.38).

### Methodological gaps

In addition to documenting the similarities and IRR between instruments, we also noted major methodological gaps in both tools. Both instruments could be improved with respect to guidance and assessment of subgroup analysis, ideally based on an a priori publicly available study protocol detailing the planned assessment of effect modification [24]. We also noted that both instruments do not consider the presentation of results using of absolute estimates of effect (e.g. risk difference for all dichotomous outcomes) [25], nor do they have an item on the overall certainty of evidence (e.g. assessed using the Grading of Recommendations Assessment, Development and Evaluation (GRADE) approach) for each outcome [26].

## Discussion

Our study aimed to compare the similarity and reliability of the AMSTAR-2 and ROBIS instruments based on 101 SR/MAs assessing nutritional interventions/exposures for cancer prevention. AMSTAR-2 is comprised of 16 items while ROBIS has 21 items, of which 12 and 14, respectively, were combined into 11 comparisons based on their conceptual similarities. Overall, we found that 70.3% (26/37) of items assess the same or similar methodological constructs. Ten comparisons were judged to fully overlap in concept and definitions, and one comparison was partially overlapping. A number of items from both tools (four in AMSTAR-2 and seven in ROBIS) were unique to each instrument and were not amenable for paired comparisons due to non-overlapping concepts, approaches, and descriptions. Both instruments do not address the reporting of absolute estimates of effect and the overall certainty of the evidence.

The study by Pieper et al. was the first to compare both instruments in terms of validity, reliability, and applicability [16]. The authors matched relevant AMSTAR-2 and ROBIS items into 12 comparisons, of which 10 were considered as fully overlapping, and two comparisons as partially overlapping (appropriateness of restriction of eligibility criteria and publication bias/robustness of the results). Our approach was similar; however, we dismissed the partially overlapping comparison between AMSTAR-2 item 3 ‘Did the review authors explain their selection of the study designs for inclusion in the review?’ and ROBIS item 1.4 ‘Were all restrictions in eligibility criteria based on study characteristics appropriate?’ as we believe these items are different constructs and are not similar enough based on underlying definitions and assessment guidance. Furthermore, while for data extraction we compared AMSTAR-2 item 6. ‘Did the review authors perform data extraction in duplicate?’ with ROBIS item 3.1 ‘Were efforts made to minimize error in data collection?’, Pieper et al. additionally considered ROBIS item 3.5 ‘Were efforts made to minimize error in risk of bias assessment?’ within this comparison. We did not include ROBIS item 3.5 into this comparison as we believe duplicate RoB assessment and duplicate data extraction should be assessed separately.

Before AMSTAR-2 was published, researchers attempted to compare the reliability of ROBIS and the original AMSTAR tool. The correlation coefficients ranged from moderate to substantial [13]. Generally, apart from one comparison of AMSTAR-2 item 8 with ROBIS item 3.2, our calculations resulted in higher coefficient values as compared to those reported by Pieper et al. [16]. Their calculated agreement levels concerning similar methodological constructs were reported to be perfect for one comparison, substantial in six comparisons, moderate in two comparisons, fair in one comparison, and slight in one comparison. By contrast, our calculations provided six items with almost perfect comparisons, three with substantial, one with a moderate, and one with a slight level of agreement. One possible explanation for these discrepancies could be the quality of included studies. In our sample of 101 articles published within the field of nutrition for cancer prevention, only 1% of SR/MAs were of high quality according to AMSTAR-2, and 3% were of low RoB according to ROBIS, which indicates mostly low scores in the majority of items of both instruments, which might result in high agreement coefficients’ values. Alternatively, unlike Pieper et al., it may be that our coefficients were higher because pairs of reviewers participated in calibration and consensus procedures, which ensured that differences in assessments were discussed, thus reducing the number of outlying assessments that might have occurred. In Pieper et al., no consensus procedure between reviewers was introduced and final judgement on items within each comparison was based on the judgments of most of the raters, resulting in the possibility of higher variation of assessments, and thus lower agreement scores.

After performing assessments using both instruments, we were surprised that both instruments did not have items devoted to the assessment of the magnitude of the effects based on absolute estimates (e.g. risk difference) for dichotomous outcomes, or the certainty of evidence for each outcome. Providing the information on these items is supported by the GRADE guidance, the Cochrane Handbook, and the Joanna Briggs Institute Manual [27–30]. Rating certainty of the evidence for each assessed health outcome improves the interpretation of SR/MA results and should be considered a vital characteristic of quality in reviews. Regarding the magnitude of the effects, authors commonly report effects as relative estimates such as risk ratios or hazard ratios while underreporting absolute measures such as the risk difference or number needed to treat [25]. Evidence suggests that reporting both relative and absolute estimates and their corresponding certainty of evidence allows for optimal interpretation of review findings [7, 25, 31–33]. Future updates of ROBIS and AMSTAR-2 instruments should consider adding these items, and interim users of the instruments might consider these items, particularly in nutrition for long term health outcomes, where the absolute effects may be small and uncertain [34–36].

We followed Cochrane guidance on systematic review methods strengthening the validity of our findings, including calibration exercises and duplicate screening, abstraction, and quality assessment. Furthermore, our methods followed an a priori study protocol and included a random sample of 101 nutrition studies, a large sample in the same healthcare field. With regard to weaknesses, first, many items in the AMSTAR-2 and ROBIS instruments were dissimilar and did not always allow for reliability comparisons, so our coefficients could be misleading to readers who may have the impression that the instruments are the same or close to the same for assessing SR/MA quality. That is, while there were many overlapping conceptual items (70.3%), there were a substantial number of dissimilar items (11/37), and so applying each instrument to the same study could result in important material differences with respect to the quality of conduct of a SR/MA. Second, while our team reported that ROBIS took longer than AMSTAR-2 assessments, we did not formally measure the time it took for reviewers to complete the assessments for each instrument. Comparisons have previously been reported indicating varying results, ranging from AMSTAR assessment taking slightly longer than ROBIS, to ROBIS assessment taking substantially longer than AMSTAR [13, 16, 37]. Third, we chose a random subsample of 101 of 737 identified studies, as completing assessments of all identified studies was not deemed feasible due to time constraints. Fourth, since the reliability of the majority of included studies assessed with AMSTAR-2 and ROBIS was critically low, the agreement coefficients between instruments in other fields of health care might differ from ours, particularly if there is more variability in the quality of SR/MAs or if higher quality SR/MAs are included.

## Conclusions

AMSTAR-2 and ROBIS are instruments designed to facilitate the assessment of SR/MA quality. Among the instruments, 70.3% of items address the same or similar methodological constructs. While the IRR of these items was moderate to perfect in fully overlapping comparisons, and slight in partially overlapping, there are unique methodological items that each instrument independently addresses. Further investigation based on samples of SR/MAs from different fields of medicine and health science might further elucidate similarities and discrepancies between both tools. Notably, AMSTAR-2 and ROBIS do not address the reporting of absolute estimates of effect or the overall certainty of the evidence, both of which are important for the optimal interpretation of SR/MA findings. The choice to use one or both of the instruments should depend on the aim of the investigators or users’ of the SR/MAs (i.e. overall methodological quality versus RoB assessment only) and other factors such as experience with the instrument or time constraints. It has previously been suggested that both instruments have areas for improvement [16, 37], findings that our systematic survey corroborates. One pragmatic instrument that fully considers RoB together with other methodological quality items such as the presentation of both relative and absolute estimates and the certainty of these estimates would optimally help users’ of SR/MAs better assess and interpret a reviews overall quality and importance of reported results.

## Supplementary Information

**Additional file 1.**

## Data Availability

The datasets used and/or analysed during the current study are available from the corresponding author on reasonable request.

## Abbreviations

AMSTAR-2: A Measurement Tool to Assess systematic Reviews, version 2
GRADE: Grading of Recommendations Assessment, Development, and Evaluation
IRR: Inter-rater reliability
MA: Meta-analysis
NRSI: Non-randomised studies of interventions/exposures
PRISMA: Preferred Reporting Items for Systematic Reviews and Meta-Analyses
RCT: Randomized controlled trial
ROB: Risk of bias
ROBIS: Risk of Bias in Systematic reviews
SR: Systematic review
95% CI: 95% confidence interval

## References

[1] Ioannidis JP (2016). The mass production of redundant, misleading, and conflicted systematic reviews and Meta-analyses. Milbank Q.

[2] In: Graham R, Mancher M, Miller Wolman D, Greenfield S, Steinberg E, editors. Clinical Practice Guidelines We Can Trust. Washington (DC)2011. 24983061

[3] Bastian H, Glasziou P, Chalmers I (2010). Seventy-five trials and eleven systematic reviews a day: how will we ever keep up?. PLoS Med.

[4] Mulrow CD (1994). Rationale for systematic reviews. BMJ..

[5] Lunny C, Ramasubbu C, Gerrish S, Liu T, Salzwedel DM, Puil L (2020). Impact and use of reviews and 'overviews of reviews' to inform clinical practice guideline recommendations: protocol for a methods study. BMJ Open.

[6] Pussegoda K, Turner L, Garritty C, Mayhew A, Skidmore B, Stevens A (2017). Systematic review adherence to methodological or reporting quality. Syst Rev..

[7] Zeraatkar D, Bhasin A, Morassut RE, Churchill I, Gupta A, Lawson DO (2021). Characteristics and quality of systematic reviews and meta-analyses of observational nutritional epidemiology: a cross-sectional study. Am J Clin Nutr.

[8] Pollock M, Fernandes RM, Becker LA, Featherstone R, Hartling L (2016). What guidance is available for researchers conducting overviews of reviews of healthcare interventions? A scoping review and qualitative metasummary. Syst Rev.

[9] Lunny C, Ramasubbu C, Puil L, Liu T, Gerrish S, Salzwedel DM (2021). Over half of clinical practice guidelines use non-systematic methods to inform recommendations: a methods study. PLoS One.

[10] Shea BJ, Grimshaw JM, Wells GA, Boers M, Andersson N, Hamel C (2007). Development of AMSTAR: a measurement tool to assess the methodological quality of systematic reviews. BMC Med Res Methodol.

[11] Shea BJ, Reeves BC, Wells G, Thuku M, Hamel C, Moran J (2017). AMSTAR 2: a critical appraisal tool for systematic reviews that include randomised or non-randomised studies of healthcare interventions, or both. BMJ..

[12] Whiting P, Savovic J, Higgins JP, Caldwell DM, Reeves BC, Shea B (2016). ROBIS: a new tool to assess risk of bias in systematic reviews was developed. J Clin Epidemiol.

[13] Banzi R, Cinquini M, Gonzalez-Lorenzo M, Pecoraro V, Capobussi M, Minozzi S (2018). Quality assessment versus risk of bias in systematic reviews: AMSTAR and ROBIS had similar reliability but differed in their construct and applicability. J Clin Epidemiol.

[14] Buhn S, Mathes T, Prengel P, Wegewitz U, Ostermann T, Robens S (2017). The risk of bias in systematic reviews tool showed fair reliability and good construct validity. J Clin Epidemiol.

[15] Lorenz RC, Matthias K, Pieper D, Wegewitz U, Morche J, Nocon M (2019). A psychometric study found AMSTAR 2 to be a valid and moderately reliable appraisal tool. J Clin Epidemiol.

[16] Pieper D, Puljak L, Gonzalez-Lorenzo M, Minozzi S (2019). Minor differences were found between AMSTAR 2 and ROBIS in the assessment of systematic reviews including both randomized and nonrandomized studies. J Clin Epidemiol.

[17] Wiseman MJ (2019). Nutrition and cancer: prevention and survival. Br J Nutr.

[18] Salam RA, Welch V, Bhutta ZA (2015). Systematic reviews on selected nutrition interventions: descriptive assessment of conduct and methodological challenges. BMC Nutr.

[19] Zajac J, Storman D, Swierz MJ, Koperny M, Tobola P, Staskiewicz W, et al. Are articles published as systematic reviews addressing nutritional exposures for cancer prevention trustworthy? A systematic survey of quality and risk of bias. Nutrition Reviews 2021:Accepted for publication. 10.1093/nutrit/nuab093.

[20] Gwet KL (2008). Computing inter-rater reliability and its variance in the presence of high agreement. Br J Math Stat Psychol.

[21] Gwet KL (2014). Handbook of inter-rater reliability: the definitive guide to measuring the extent of agreement among raters.

[22] Landis JRKG (1977). The measurement of observer agreement for categorical data. Biometrics.

[23] Page MJ, McKenzie JE, Bossuyt PM, Boutron I, Hoffmann TC, Mulrow CD (2021). The PRISMA 2020 statement: an updated guideline for reporting systematic reviews. BMJ..

[24] Schandelmaier S, Briel M, Varadhan R, Schmid CH, Devasenapathy N, Hayward RA (2020). Development of the instrument to assess the credibility of effect modification analyses (ICEMAN) in randomized controlled trials and meta-analyses. CMAJ..

[25] Alonso-Coello P, Carrasco-Labra A, Brignardello-Petersen R, Neumann I, Akl EA, Vernooij RW (2016). Systematic reviews experience major limitations in reporting absolute effects. J Clin Epidemiol.

[26] Guyatt GH, Oxman AD, Vist GE, Kunz R, Falck-Ytter Y, Alonso-Coello P (2008). GRADE: an emerging consensus on rating quality of evidence and strength of recommendations. BMJ..

[27] Schünemann HJ, Higgins JPT, Vist GE, Glasziou P, Akl EA, Skoetz N, et al. chapter 14: completing ‘summary of findings’ tables and grading the certainty of the evidence. In: Higgins JPT, Thomas J, Chandler J, Cumpston M, li T, Page MJ, Welch VA (editors) Cochrane handbook for systematic reviews of interventions version 62 (updated February 2021) Cochrane, 2021. Available from www.training.cochrane.org/handbook.

[28] Santesso N, Glenton C, Dahm P, Garner P, Akl EA, Alper B (2020). GRADE guidelines 26: informative statements to communicate the findings of systematic reviews of interventions. J Clin Epidemiol.

[29] Langendam MW, Akl EA, Dahm P, Glasziou P, Guyatt G, Schunemann HJ (2013). Assessing and presenting summaries of evidence in Cochrane reviews. Syst Rev..

[30] Aromataris E (2020). Munn Z, (editors).

[31] Agarwal A, Johnston BC, Vernooij RW, Carrasco-Labra A, Brignardello-Petersen R, Neumann I (2017). Authors seldom report the most patient-important outcomes and absolute effect measures in systematic review abstracts. J Clin Epidemiol.

[32] Akl EA, Oxman AD, Herrin J, Vist GE, Terrenato I, Sperati F (2011). Using alternative statistical formats for presenting risks and risk reductions. Cochrane Database Syst Rev.

[33] Neumann I, Alonso-Coello P, Vandvik PO, Agoritsas T, Mas G, Akl EA (2018). Do clinicians want recommendations? A multicenter study comparing evidence summaries with and without GRADE recommendations. J Clin Epidemiol.

[34] Han MA, Zeraatkar D, Guyatt GH, Vernooij RWM, El Dib R, Zhang Y (2019). Reduction of red and processed meat intake and Cancer mortality and incidence: a systematic review and Meta-analysis of cohort studies. Ann Intern Med.

[35] Vernooij R, Guyatt GH, Zeraatkar D, Han MA, Valli C, El Dib R, et al. Reconciling contrasting guideline recommendations on red and processed meat for health outcomes. J Clin Epidemiol. 2021. 10.1016/j.jclinepi.2021.07.008. 10.1016/j.jclinepi.2021.07.008 34273525

[36] Johnston BC, Zeraatkar D, Han MA, Vernooij RWM, Valli C, El Dib R (2019). Unprocessed red meat and processed meat consumption: dietary guideline recommendations from the nutritional recommendations (NutriRECS) consortium. Ann Intern Med.

[37] Gates M, Gates A, Duarte G, Cary M, Becker M, Prediger B (2020). Quality and risk of bias appraisals of systematic reviews are inconsistent across reviewers and centers. J Clin Epidemiol.

