# Supplementary material for: Similarities, reliability and gaps in assessing the quality of conduct of systematic reviews using AMSTAR-2 and ROBIS: systematic survey of nutrition reviews
Source: BMC Med Res Methodol. 2021 Nov 27;21:261. doi: 10.1186/s12874-021-01457-w (PMC8627612; doi:10.1186/s12874-021-01457-w)
Supplement: Supplementary file 1 — Additional file 1. [file 12874_2021_1457_MOESM1_ESM.docx]

Supplementary file: Search strategies

OVID MEDLINE

1. (embase or cinhal or cinahl or amed or psychlit or psyclit or psychinfo or psycinfo or science).tw. and (citation and index).mp.
2. (cochrane or cancerlit or aicr or iarc or epic or uspstf).tw.
3. reference.mp. and list*.ab. [mp=title, abstract, original title, name of substance word, subject heading word, floating sub-heading word, keyword heading word, protocol supplementary concept word, rare disease supplementary concept word, unique identifier, synonyms]
4. (selection.mp. and criteria.ab.) or (data and extraction.ab. and review).hw,tw. [mp=title, abstract, original title, name of substance word, subject heading word, floating sub-heading word, keyword heading word, protocol supplementary concept word, rare disease supplementary concept word, unique identifier, synonyms]
5. exp meta analysis/ or meta analysis.tw. or systematic* review*.tw. or systematic* overview*.tw. or exp literature/
6. ((cancer prevention or oncoprevention or chemoprevention).hw,tw. or exp chemoprevention/ or cancer chemoprevention.hw,tw. or chemoprophylaxis.hw,tw. or chemoprophylaxis.mp. or chemopreventive.hw,tw. or malignan*.tw. or oncogen*.tw. or cancerogen*.tw. or adeno*.tw. or tumo$r*.tw. or carcinom*.tw. or cancer*.tw. or neoplasm*.tw. or oncol*.tw. or exp neoplasm/ or exp leukemia/ or leukemia.tw. or leukaemia.tw. or leukemias.tw. or leukaemias.tw. or leucocythaemia.tw. or hematologic malignancy.tw. or haematologic malignancy.tw. or hematologic neoplasms.tw. or absarcoma.tw. or blastoma.tw. or myelodysplastic syndrome.tw. or myelodysplastic syndromes.tw. or transient myeloproliferative disorder.tw. or hodgkin disease.tw. or hodgkins disease.tw. or carcinoma.tw.) not (radiation therapy.tw. or exp radiotherapy/ or (radiotherapy or treatment associated cancer or chemotherapy or pharmacotherapy).tw. or cancer immunotherapy.tw. or cancer surgery.tw. or molecularly targeted therapy.tw.).hw,tw. [mp=title, abstract, original title, name of substance word, subject heading word, floating sub-heading word, keyword heading word, protocol supplementary concept word, rare disease supplementary concept word, unique identifier, synonyms]
7. 1 or 2 or 3 or 4 or 5
8. (comment or letter or editorial or (animals not (animals and human))).hw,tw.
9. (((((embase or cinhal or cinahl or amed or psychlit or psyclit or psychinfo or psycinfo or science).tw. and (citation and index).mp.) or (cochrane or cancerlit or aicr or iarc or epic or uspstf).tw. or reference.mp.) and list*.ab.) or (selection.mp. and criteria.ab.) or (data and extraction.ab. and review).hw,tw. or exp meta analysis/ or meta analysis.tw. or systematic* review*.tw. or systematic* overview*.tw. or exp literature/) not (comment or letter or editorial or (animals not (animals and human))).hw,tw.
10. 6 and 9
11. exp alpha tocopherol/ or alpha tocopherol.tw. or tocopherol.tw. or vitamin e.tw. or betacarotene*.tw. or (beta and carotene*).tw. or carotene*.tw. or carotenoid*.tw. or caroten*.tw. or exp retinoic acid/ or retinoic acid*.tw. or retinoid*.tw. or tretinoin.tw. or vitamin a acid.tw. or trans-retinoic acid.tw. or trans retinoic acid.tw. or all-trans-retinoic acid.tw. or all trans retinoic acid.tw. or beta-all-trans-retinoic acid.tw. or beta all trans retinoic acid.tw. or 3-cis-ra.tw. or 13-cis-retinoic acid.tw. or retin-a.tw. or retin a.tw. or isotretinoin.tw. or exp folic acid/ or folic acid.tw. or folate.tw. or vitamin m.tw. or folacin.tw. or pteroylglutamic acid.tw. or exp iron/ or iron*.tw. or exp magnesium/ or mgso4.tw. or magnesium sulfate.tw. or magnesum.tw. or exp magnesium sulfate/ or exp trace element/ or trace element.tw. or trace elements.tw. or micronutrient*.tw. or exp dietary supplement/ or dietary supplement.tw. or mineral supplement.tw. or mineral supplements.tw. or antioxidant*.tw. or vitamin*.tw. or probiotic*.tw. or exp probiotic/ or exp synbiotic/ or synbiotic*.tw. or exp lactobacillus/ or lactobacil*.tw. or exp bifidobacterium/ or bifidobacterium.tw. or bifidus.tw. or bifidobacter*.tw. or streptococc*.tw. or lactococc*.tw. or leuconostoc.tw. or pediococc*.tw. or beneficial bacteria.tw. or exp selenium/ or selen*.tw. or selenium derivative.tw. or methylseleninic acid.tw. or methylselenium.tw. or organoselenium derivative.tw. or exp sodium selenite/ or sodium selenite.tw. or exp retinol/ or retinol*.tw. or retinal*.tw. or aquasol a.tw. or vitamin a.tw. or exp vitamin b complex/ or exp thiamine/ or thiamin*.tw. or niacin*.tw. or cobalamin*.tw. or biotin*.tw. or neurobion*.tw. or pantothenic acid.tw. or pyridox.tw. or exp pyridoxine/ or benfotiamine.tw. or pyridoxine.tw. or vitamin b*.tw. or vitamin b1.tw. or vitamin b 1.tw. or vitamin b2.tw. or vitamin b 2.tw. or vitamin b3.tw. or vitamin b 3.tw. or vitamin b5.tw. or vitamin b 5.tw. or vitamin b6.tw. or vitamin b 6.tw. or vitamin b7.tw. or vitamin b 7.tw. or vitamin b9.tw. or vitamin b 9.tw. or vitamin b12.tw. or vitamin b 12.tw. or exp ascorbic acid/ or ascorb*.tw. or exp vitamin d/ or vitamin d.tw. or vitamin d2.tw. or vitamin d 2.tw. or vitamin d3.tw. or vitamin d 3.tw. or calciol.tw. or exp ergocalciferol/ or ergocalciferol*.tw. or exp cholecalciferol/ or cholecalciferol.tw. or cholecalciferol.tw. or hydroxycholecalciferol.tw. or calcitriol.tw. or dihydroxyvitamin d3.tw. or alphacalcidol.tw. or beta tocopherol.tw. or gamma tocopherol.tw. or exp riboflavin/ or riboflavin*.tw. or vitamin g.tw. or exp molybdenum/ or molybdenum.tw. or exp selenomethionine/ or selenomethionine.tw. or exp zinc/ or zinc.tw. or exp oxidizing agent/ or antioxidant*.tw. or anti oxidant*.tw. or exp calcium/ or calcium.tw. or exp calcium carbonate/ or calcium carbonate.tw. or exp vitamin k/ or exp phytomenadione/ or phytomenadione.tw. or phytonadione.tw. or phylloquinone.tw. or phylloquinine.tw. or phyllohydroquinone.tw. or aquamephyton.tw. or menaquinone*.tw. or menadione.tw. or 2 methyl 14 naphthoquinone.tw. or 2 methylnaphthoquinone.tw. or 2 methyl 14 naphthalenedione.tw. or acetomenaphthone.tw. or farnoquinone.tw. or menadiol.tw. or menatetrenone.tw. or vitamin k.tw. or macronutrient*.tw.
12. exp fish oil/ or (fish and oil*).tw. or exp cod liver oil/ or cod liver oil.tw. or exp omega 3 fatty acid/ or omega 3 fatty acid.tw. or vegetable oil*.tw. or plant oil*.tw. or exp butter/ or butter.tw. or fat intake.tw. or edible oil*.tw. or fat*.tw. or (fatty and acid*).tw. or monounsaturated fatty acid*.tw. or monounsaturated fat*.tw. or mufa.tw. or docosahexaenoic acid.tw. or dha.tw. or pufa.tw. or eicosapentanoic acid.tw. or epa.tw. or (volatile and fatty and acid*).tw. or volatile fatty acid.tw. or exp margarine/ or margarine.tw. or oil*.tw. or olive tree.tw. or exp olive oil/ or olive.tw. or olea.tw. or exp oleic acid/ or oleic acid.tw. or perilla seed oil.tw. or perilla oil.tw. or exp thioctic acid/ or exp lipoic acid/ or lipoic acid.tw.
13. exp plant/ or exp vegetable/ or exp vegetable oil/ or exp fruit/ or plant*.tw. or vegetable*.tw. or vegetable oil.tw. or fruit*.tw. or exp anthocyanin/ or anthocyanin*.tw. or apple*.tw. or banan*.tw. or bean*.tw. or berry.tw. or brassica.tw. or broccoli.tw. or (brussel* and sprout*).tw. or cabbage*.tw. or carrot*.tw. or citrus.tw. or cranberr*.tw. or cucumber*.tw. or dry bean.tw. or epigallocatechol.tw. or gallocatechin.tw. or gallocatechol.tw. or dietary fib*.tw. or starch.tw. or high fib*.tw. or high-fiber.tw. or fiber.tw. or fibre.tw. or exp starch/ or flavonoid.tw. or flavanone*.tw. or flavonoid*.tw. or bioflavonoid*.tw. or flav$none*.tw. or flavone*.tw. or flav$nol*.tw. or flavanolignan*.tw. or quercetin.tw. or rutin*.tw. or oxerutin*or.tw. or kaempferol.tw. or myricetin.tw. or apigenin.tw. or luteolin.tw. or anthocyanidin.tw. or catechin.tw. or epicatechin.tw. or naringenin.tw. or hesperetin.tw. or daidzein.tw. or genistein.tw. or hydroxyethylrutoside*.tw. or o s hydroxyethyl rutoside*.tw. or (b hydroxyethyl and rutoside*).tw. or catechin*.tw. or (procyanidolic and oligomers).tw. or flavon 3 ol*.tw. or benzoflavone*.tw. or flavanolignan*.tw. or flav$n*.tw. or isoflavone*.tw. or epigallocatechin gallate.tw. or propyl gallate.tw. or gallic acid propyl ester.tw. or fruit juice*.tw. or vegetable juice*.tw. or juice*.tw. or garlic.tw. or ginger*.tw. or exp spice/ or spice*.tw. or tomato*.tw. or lycopene.tw. or onion*.tw. or palmetto.tw. or phytoestrogen*.tw. or plant estrogen.tw. or exp phytoestrogen/ or polyphenol*.tw. or punicaceae.tw. or pomegranate*.tw. or potato*.tw. or cucurbita.tw. or pumpkin.tw. or soybean*.tw. or soy food*.tw. or tofu.tw. or daidzin*.tw. or daidzein*.tw. or genistin*.tw. or genistein*.tw. or glycetin*.tw. or glycetein*.tw. or miso.tw. or soy food.tw. or spinach.tw. or strawberr*.tw. or sweet potato.tw. or sweet potatos.tw. or ipomoea batatas.tw. or chive*.tw. or flax*.tw. or flaxseed*.tw. or linseed*.tw. or oilseed.tw. or mushroom*.tw. or fungi.tw. or ginkgo biloba extract.tw. or exp ginkgo/ or ginkgo*.tw. or gingko*.tw. or gingkco.tw. or ginko*.tw. or gingho.tw. or gincosan.tw. or bilobalid*.tw. or tebonin.tw. or tebokan.tw. or kaveri*.tw. or tanakan.tw. or tanakene.tw. or rokan.tw. or supergin*.tw. or egb*.tw. or isothiocyanate*.tw. or isothiocyanic acid.tw. or exp seaweed/ or kelp.tw. or exp panax/ or ginseng.tw. or seed plant.tw. or seed*.tw. or leek*.tw. or exp bread/ or bread*.tw. or exp sorghum/ or sorghum.tw. or exp cereal/ or cereal*.tw. or food grain.tw. or corn.tw. or cornmeal.tw. or cornmeal*.tw. or bulgar.tw. or bulghar.tw. or couscous*.tw. or porridge.tw. or rice*.tw. or exp rice/ or millet.tw. or maize*.tw. or barley*.tw. or rye*.tw. or oat*.tw. or exp whole grain/ or wholegrain*.tw. or wholemeal*.tw. or wholewheat*.tw. or (whole adj3 grain*).tw. or (whole adj3 wheat).tw. or (whole adj3 food).tw. or (wheat adj3 meat).tw. or grain*.tw. or wheat*.tw.
14. meat*.tw. or exp meat/ or exp meat products/ or exp red meat/ or red meat.tw. or processed meat.tw. or pork.tw. or exp pork/ or exp poultry/ or poultry.tw. or sausage.tw. or animal* protein*.tw. or bacon*.tw. or exp beef/ or beef.tw. or exp chicken/ or chicken*.tw. or lard.tw.
15. exp skate fish/ or exp fish product/ or exp fish protein/ or fish*.tw. or exp sea food/ or sea food.tw. or seafood.tw. or exp shellfish/ or surimi.tw.
16. exp ethanol/ or ethanol.tw. or alcohol.tw. or exp beer/ or beer.tw. or caffeine.tw. or exp caffeine/ or caffeine.tw. or exp catechin/ or catechin.tw. or (catechinic and acid).tw. or cianidanol*.tw. or ciandiol*.tw. or cianidol*.tw. or cyanidanol*.tw. or exp coffea/ or coffea.tw. or exp coffee/ or coffee.tw. or coffe*.tw. or exp tea/ or tea.tw. or teas.tw. or decaffeinated tea.tw. or ((tea or teas) adj3 extract*).tw. or camellia sinensis.tw. or exp drinking behavior/ or exp wine/ or wine.tw. or resveratrol.tw. or (resvida or longivenex or polygonum cuspidatum or resv or srt 501 or srt501 or trans resveratrol).tw. or (red and wine).tw. or stilbene.tw. or japanese knotweed.tw. or decaffeinated coffee.tw.
17. exp cheese/ or cheese.tw. or che$se*.tw. or exp dairy product/ or (dairy and food).tw. or (dairy and product*).tw. or exp milk/ or exp milk proteins/ or exp cultured milk products/ or ((milk or cream*) adj2 (ferment* or cultur* or product* or sour* or protein*)).tw. or exp yoghurt/ or yogourt.tw. or yogurt.tw. or yoghurt.tw. or kefir.tw. or buttermilk.tw. or exp lactose/ or glucose.tw. or anhydrous lactose.tw.
18. 11 or 12 or 13 or 14 or 15 or 16 or 17
19. 6 and 9 and 18

Embase:

(((embase:ab,ti OR cinhal:ab,ti OR cinahl:ab,ti OR amed:ab,ti OR psychlit:ab,ti OR psyclit:ab,ti OR psychinfo:ab,ti OR psycinfo:ab,ti OR science:ab,ti) AND citation:ab,ti AND index:ab,ti OR cochrane:ab,ti OR cancerlit:ab,ti OR aicr:ab,ti OR iarc:ab,ti OR epic:ab,ti OR uspstf:ab,ti OR (reference AND list*.:ab) OR bibliograph*:ab OR (relevant AND journals:ab) OR (manual AND search*:ab) OR ((selection AND criteria:ab OR (data AND extraction:ab)) AND review) OR 'meta analysis (topic)'/exp OR 'meta analysis' OR (systematic*:ti,ab AND (review*:ti,ab OR overview*:ti,ab)) OR 'literature'/exp) NOT (comment OR letter OR editorial OR (animals NOT (animals AND human))) AND (malignan*:ti,ab OR oncogen*:ti,ab OR cancerogen*:ti,ab OR adeno*:ti,ab OR tumo?r*:ti,ab OR carcinom*:ti,ab OR cancer*:ti,ab OR neoplasm*:ti,ab OR oncol*:ti,ab OR 'oncology'/exp OR 'neoplasm'/exp OR 'leukemia'/exp OR leukemia:ab,ti OR leukaemia:ab,ti OR leukemias:ab,ti OR leukaemias:ab,ti OR leucocythaemia:ab,ti OR 'hematologic malignancy':ab,ti OR 'haematologic malignancy':ab,ti OR 'hematologic neoplasms':ab,ti OR absarcoma:ab,ti OR blastoma:ab,ti OR 'myelodysplastic syndrome':ab,ti OR 'myelodysplastic syndromes':ab,ti OR 'transient myeloproliferative disorder':ab,ti OR 'hodgkin disease':ab,ti OR 'hodgkins disease':ab,ti OR carcinoma:ab,ti)) AND ((('cheese'/exp OR cheese OR che?se* OR 'dairy product'/exp OR (dairy AND food:ab,ti) OR (dairy AND product*:ab,ti) OR 'milk'/exp OR 'milk proteins'/exp OR 'cultured milk products'/exp OR (((milk OR cream*) NEAR/2 (ferment* OR cultur* OR product* OR sour* OR protein*)):ab,ti) OR 'yoghurt'/exp OR yogourt:ab,ti OR yogurt:ab,ti OR yoghurt:ab,ti OR kefir:ab,ti OR buttermilk:ab,ti OR 'lactose'/exp OR 'd glucose':ab,ti OR 'anhydrous lactose':ab,ti) OR ('ethanol'/exp OR ethanol:ab,ti OR 'alcohol'/exp OR alcohol*:ab,ti OR 'beer'/exp OR beer:ab,ti OR caffeine:ab,ti OR 'caffeine'/exp OR caffeine OR 'catechin'/exp OR 'catechin' OR (catechinic AND acid:ab,ti) OR cianidanol*:ab,ti OR ciandiol*:ab,ti OR cianidol*:ab,ti OR cyanidanol*:ab,ti OR 'coffea'/exp OR 'coffea':ab,ti OR 'coffee'/exp OR coffee OR coffe*:ab,ti OR 'tea'/exp OR tea OR tea:ab,ti OR teas:ab,ti OR 'decaffeinated tea':ab,ti OR (((tea OR teas) NEAR/3 extract*):ab,ti) OR 'camellia sinensis':ab,ti OR 'drinking behavior'/exp OR 'wine'/exp OR wine:ab,ti OR 'resveratrol'/exp OR (((resvida OR longivenex OR 'polygonum cuspidatum' OR resv OR 'srt 501' OR srt) AND 501 OR srt501 OR 'trans resveratrol' OR red) AND wine) OR stilbene OR 'japanese knotweed':ab,ti OR 'decaffeinated coffee':ab,ti) OR ('skate (fish)'/exp OR 'fish product'/exp OR 'fish protein'/exp OR fish*:ab,ti OR 'fish'/exp OR 'sea food'/exp OR 'sea food':ab,ti OR 'seafood':ab,ti OR 'shellfish'/exp OR 'surimi'/exp OR 'surimi':ab,ti) OR (meat*:ab,ti OR 'meat'/exp OR 'meat products'/exp OR 'red meat'/exp OR 'red meat':ab,ti OR 'processed meat':ab,ti OR 'processed meat'/exp OR 'pork':ab,ti OR 'poultry':ab,ti OR 'sausage':ab,ti OR 'animal protein'/exp OR 'animal proteins':ab,ti OR 'animals proteins':ab,ti OR 'animals protein':ab,ti OR bacon*:ab,ti OR 'beef'/exp OR 'beef':ab,ti OR 'chicken'/exp OR chicken*:ab,ti OR 'lard'/exp OR 'lard':ab,ti) OR ('bread'/exp OR 'bread*':ab,ti OR 'sorghum'/exp OR 'sorghum (genus)'/exp OR sorghum:ab,ti OR 'cereal'/exp OR 'bakery product'/exp OR 'food grain'/exp OR cereal*:ab,ti OR 'food grain':ab,ti OR corn:ab,ti OR cornmeal:ab,ti OR cornmeal*:ab,ti OR (corn AND meals:ab,ti) OR bulgar:ab,ti OR bulghar:ab,ti OR couscous*:ab,ti OR porridge:ab,ti OR rice*:ab,ti OR 'rice'/exp OR millet:ab,ti OR maize*:ab,ti OR barley*:ab,ti OR rye*:ab,ti OR oat*:ab,ti OR 'whole grain'/exp OR wholegrain*:ab,ti OR wholemeal*:ab,ti OR wholewheat*:ab,ti OR ((whole NEAR/3 grain*):ab,ti) OR ((whole NEAR/3 wheat):ab,ti) OR ((whole NEAR/3 food):ab,ti) OR ((wheat NEAR/3 meat):ab,ti) OR 'grain*':ab,ti OR 'wheat*':ab,ti) OR ('plant'/exp OR 'vegetable'/exp OR 'vegetable oil'/exp OR 'fruit'/exp OR plant*:ab,ti OR vegetable*:ab,ti OR 'vegetable oil':ab,ti OR fruit*:ab,ti OR 'plant medicinal product'/exp OR 'anthocyanin'/exp OR anthocyanin*:ab,ti OR apple*:ab,ti OR banan*:ab,ti OR bean*:ab,ti OR berry:ab,ti OR brassica:ab,ti OR broccoli:ab,ti OR (brussels:ab,ti AND sprout:ab,ti) OR (brussels:ab,ti AND sprouts:ab,ti) OR cabbage*:ab,ti OR carrot*:ab,ti OR citrus:ab,ti OR cranberr*:ab,ti OR 'fruit vegetable'/exp OR cucumber*:ab,ti OR 'dry bean':ab,ti OR epigallocatechol:ab,ti OR gallocatechin:ab,ti OR gallocatechol:ab,ti OR 'dietary fiber'/exp OR 'dietary fiber':ab,ti OR 'dietary fibre':ab,ti OR 'starch':ab,ti OR 'high fiber':ab,ti OR 'high-fiber':ab,ti OR fiber:ab,ti OR 'starch'/exp OR 'flavonoid'/exp OR flavonoid:ab,ti OR 'flavanone derivative'/exp OR flavanone*:ab,ti OR 'isoflavone derivative'/exp OR flavonoid*:ab,ti OR bioflavonoid*:ab,ti OR flav?none*:ab,ti OR flavone*:ab,ti OR flav?nol*:ab,ti OR flavanolignan*:ab,ti OR (((((quercetin:ab,ti OR rutin*:ab,ti OR oxerutin*or:ab,ti) AND kaempferol:ab,ti OR myricetin:ab,ti OR apigenin:ab,ti OR luteolin:ab,ti OR anthocyanidin:ab,ti OR catechin:ab,ti OR epicatechin:ab,ti OR naringenin:ab,ti OR hesperetin:ab,ti OR daidzein:ab,ti OR genistein:ab,ti OR hydroxyethylrutoside*:ab,ti OR 'o s hydroxyethyl rutoside*':ab,ti OR 'b hydroxyethyl':ab,ti) AND rutoside*:ab,ti OR catechin*:ab,ti OR procyanidolic:ab,ti) AND oligomers:ab,ti OR 'flavon 3 ol*':ab,ti OR benzoflavone*or:ab,ti) AND flavanolignan*:ab,ti) OR flav?n*:ab,ti OR isoflavone*:ab,ti OR 'epigallocatechin gallate':ab,ti OR 'propyl gallate':ab,ti OR 'gallic acid propyl ester'/exp OR 'fruit juice':ab,ti OR 'vegetable juice':ab,ti OR 'fruit juices':ab,ti OR 'vegetable juices':ab,ti OR juice*:ab,ti OR garlic:ab,ti OR ginger*:ab,ti OR 'spice'/exp OR spice*:ab,ti OR tomato*:ab,ti OR lycopene:ab,ti OR onion*:ab,ti OR palmetto:ab,ti OR phytoestrogen*:ab,ti OR 'plant estrogen':ab,ti OR 'phytoestrogen'/exp OR polyphenol*:ab,ti OR 'polyphenol'/exp OR punicaceae:ab,ti OR pomegranate*:ab,ti OR potato*:ab,ti OR cucurbita:ab,ti OR pumpkin:ab,ti OR soybean*:ab,ti OR 'soy food':ab,ti OR tofu:ab,ti OR daidzin*:ab,ti OR daidzein*:ab,ti OR genistin*:ab,ti OR genistein*:ab,ti OR glycetin*:ab,ti OR glycetein*:ab,ti OR miso:ab,ti OR 'soy food'/exp OR spinach:ab,ti OR strawberr*:ab,ti OR 'sweet potato':ab,ti OR 'sweet potatos':ab,ti OR 'ipomoea batatas':ab,ti OR chive*:ab,ti OR 'flax'/exp OR flax*:ab,ti OR flaxseed*:ab,ti OR linseed*:ab,ti OR 'oilseed'/exp OR 'mushrooms'/exp OR 'mushroom*':ab,ti OR 'fungi':ab,ti OR 'ginkgo biloba extract'/exp OR 'ginkgo'/exp OR ginkgo*:ab,ti OR gingko*:ab,ti OR gingkco:ab,ti OR ginko*:ab,ti OR gingho:ab,ti OR gincosan:ab,ti OR bilobalid*:ab,ti OR tebonin:ab,ti OR tebokan:ab,ti OR kaveri*:ab,ti OR tanakan:ab,ti OR tanakene:ab,ti OR rokan:ab,ti OR supergin*:ab,ti OR egb*:ab,ti OR isothiocyanate*:ab,ti OR 'isothiocyanic acid'/exp OR 'seaweed'/exp OR kelp:ab,ti OR 'panax'/exp OR ginseng:ab,ti OR 'seed plant'/exp OR seed*:ab,ti OR leek*:ab,ti) OR ('fish oil'/exp OR (fish:ab,ti AND oil*:ab,ti) OR 'cod liver oil'/exp OR 'cod liver oil':ab,ti OR 'omega 3 fatty acid'/exp OR 'omega 3 fatty acid':ab,ti OR 'vegetable oil'/exp OR 'plant oil':ab,ti OR 'plant oils':ab,ti OR 'butter'/exp OR butter:ab,ti OR 'fat intake'/exp OR 'edible oil'/exp OR 'fat'/exp OR fat*:ab,ti OR 'fatty acid'/exp OR (fatty:ab,ti AND acid*:ab,ti) OR 'monounsaturated fatty acid'/exp OR 'monounsaturated fat':ab,ti OR mufa:ab,ti OR 'docosahexaenoic acids':ab,ti OR 'docosahexaenoic acid'/exp OR dha:ab,ti OR pufa:ab,ti OR 'eicosapentanoic acid'/exp OR 'eicosapentanoic acid':ab,ti OR 'eicosapentanoic acids':ab,ti OR epa:ab,ti OR (volatile:ab,ti AND fatty:ab,ti AND acid*:ab,ti) OR 'volatile fatty acid'/exp OR 'margarine'/exp OR margarine:ab,ti OR 'oil'/exp OR oil*:ab,ti OR 'olive tree'/exp OR 'olive oil'/exp OR olive:ab,ti OR olea:ab,ti OR 'oleic acid'/exp OR 'oleic acid':ab,ti OR 'perilla seed oil':ab,ti OR 'perilla oil':ab,ti OR 'thioctic acid'/exp OR 'lipoic acid':ab,ti) OR ('alpha tocopherol'/exp OR 'alpha tocopherol':ab,ti OR 'tocopherol':ab,ti OR 'vitamin e':ab,ti OR betacarotene*:ab,ti OR (beta:ab,ti AND carotene*:ab,ti) OR carotene:ab,ti OR carotenoid*:ab,ti OR caroten*:ab,ti OR 'retinoic acid'/exp OR 'retinoic acid':ab,ti OR 'retinoic acids':ab,ti OR retinoid*:ab,ti OR tretinoin:ab,ti OR 'vitamin a acid':ab,ti OR 'trans-retinoic acid':ab,ti OR 'trans retinoic acid':ab,ti OR 'all-trans-retinoic acid':ab,ti OR 'all trans retinoic acid':ab,ti OR 'beta-all-trans-retinoic acid':ab,ti OR 'beta all trans retinoic acid':ab,ti OR '3-cis-ra':ab,ti OR '13-cis-retinoic acid':ab,ti OR 'retin-a':ab,ti OR 'retin a':ab,ti OR 'isotretinoin':ab,ti OR 'folic acid'/exp OR 'folic acid':ab,ti OR 'folate':ab,ti OR 'vitamin m':ab,ti OR 'folacin':ab,ti OR 'pteroylglutamic acid':ab,ti OR 'iron'/exp OR iron*:ab,ti OR 'magnesium'/exp OR mgso4:ab,ti OR 'magnesium sulfate':ab,ti OR 'magnesum':ab,ti OR 'magnesium sulfate'/exp OR 'trace element'/exp OR 'trace element':ab,ti OR 'trace elements':ab,ti OR micronutrient*:ab,ti OR 'dietary supplement'/exp OR 'dietary supplement':ab,ti OR 'mineral supplementation'/exp OR 'mineral supplement':ab,ti OR 'mineral supplements':ab,ti OR 'antioxidant'/exp OR 'vitamin'/exp OR vitamin*:ab,ti OR 'probiotic agent'/exp OR probiotic*:ab,ti OR 'synbiotic agent'/exp OR synbiotic*:ab,ti OR 'lactobacillus'/exp OR lactobacil*:ab,ti OR 'bifidobacterium'/exp OR 'bifidobacterium':ab,ti OR bifidus:ab,ti OR bifidobacter*:ab,ti OR streptococc*:ab,ti OR lactococc*:ab,ti OR leuconostoc:ab,ti OR pediococc*:ab,ti OR 'beneficial bacteria':ab,ti OR 'selenium'/exp OR selen*:ab,ti OR 'selenium derivative'/exp OR 'selenium derivative':ab,ti OR 'methylseleninic acid':ab,ti OR 'methylselenium':ab,ti OR 'organoselenium derivative'/exp OR 'sodium selenite'/exp OR 'sodium selenite':ab,ti OR 'retinol'/exp OR retinol*:ab,ti OR retinal*:ab,ti OR 'aquasol a':ab,ti OR 'vitamin a':ab,ti OR 'vitamin b complex'/exp OR 'thiamine'/exp OR thiamin*:ab,ti OR niacin*:ab,ti OR cobalamin*:ab,ti OR biotin*:ab,ti OR neurobion*:ab,ti OR 'pantothenic acid':ab,ti OR 'pyridox':ab,ti OR 'pyridoxine'/exp OR 'benfotiamine'/exp OR 'benfotiamine':ab,ti OR 'pyridoxine':ab,ti OR 'vitamin b group'/exp OR 'vitamin b1':ab,ti OR 'vitamin b 1':ab,ti OR 'vitamin b2':ab,ti OR 'vitamin b 2':ab,ti OR 'vitamin b3':ab,ti OR 'vitamin b 3':ab,ti OR 'vitamin b5':ab,ti OR 'vitamin b 5':ab,ti OR 'vitamin b6':ab,ti OR 'vitamin b 6':ab,ti OR 'vitamin b7':ab,ti OR 'vitamin b 7':ab,ti OR 'vitamin b9':ab,ti OR 'vitamin b 9':ab,ti OR 'vitamin b12':ab,ti OR 'vitamin b 12':ab,ti OR 'ascorbic acid'/exp OR ascorb*:ab,ti OR 'vitamin d'/exp OR 'vitamin d':ab,ti OR 'vitamin d2':ab,ti OR 'vitamin d 2':ab,ti OR 'vitamin d3':ab,ti OR 'vitamin d 3':ab,ti OR calciol:ab,ti OR 'ergocalciferol'/exp OR ergocalciferol*:ab,ti OR 'colecalciferol'/exp OR 'colecalciferol':ab,ti OR 'cholecalciferol':ab,ti OR 'hydroxycholecalciferol':ab,ti OR 'calcitriol':ab,ti OR 'dihydroxyvitamin d3':ab,ti OR 'alphacalcidol':ab,ti OR 'beta tocopherol':ab,ti OR 'gamma tocopherol':ab,ti OR 'riboflavin'/exp OR riboflavin*:ab,ti OR 'vitamin g':ab,ti OR 'molybdenum'/exp OR 'molybdenum':ab,ti OR 'selenomethionine'/exp OR 'selenomethionine':ab,ti OR 'zinc'/exp OR 'zinc':ab,ti OR 'oxidizing agent'/exp OR antioxidant*:ab,ti OR 'anti oxidant*':ab,ti OR 'calcium'/exp OR calcium:ab,ti OR ('calcium'/exp AND 'carbonate'/exp) OR 'calcium carbonate':ab,ti OR 'vitamin k group'/exp OR 'phytomenadione'/exp OR phytomenadione:ab,ti OR phytonadione:ab,ti OR phylloquinone:ab,ti OR phylloquinine:ab,ti OR phyllohydroquinone:ab,ti OR aquamephyton:ab,ti OR menaquinone*:ab,ti OR menadione:ab,ti OR '2 methyl 14 naphthoquinone':ab,ti OR '2 methylnaphthoquinone':ab,ti OR '2 methyl 14 naphthalenedione':ab,ti OR 'acetomenaphthone':ab,ti OR farnoquinone:ab,ti OR menadiol:ab,ti OR menatetrenone:ab,ti OR 'vitamin k':ab,ti OR 'macronutrient'/exp OR 'macronutrient*':ab,ti)) OR ('diet therapy'/exp OR 'diet therapy':ab,ti OR food*:ab,ti OR 'food'/exp OR 'dietary intake'/exp OR 'dietary intake':ab,ti OR 'diet'/exp OR diet*:ab,ti OR dietetic OR dietary OR beverage*:ab,ti OR 'herb'/exp OR herb*:ab,ti))

Cochrane:

#1 (che?se):ti,ab,kw OR (cheese*):ti,ab,kw OR (cheese):ti,ab,kw OR ("dairy product"):ti,ab,kw OR ("dairy products"):ti,ab,kw OR ("dairy food"):ti,ab,kw or (milk):ti,ab,kw OR ('milk proteins'):ti,ab,kw OR ('cultured milk products'):ti,ab,kw OR (yoghurt):ti,ab,kw OR (yogurt):ti,ab,kw OR (yogourt):ti,ab,kw OR (kefir):ti,ab,kw OR (buttermilk):ti,ab,kw OR (lactose):ti,ab,kw OR (“d glucose”):ti,ab,kw OR (“anhydrous lactose”):ti,ab,kw

#2 MeSH descriptor: [Cheese] explode all trees

#3 MeSH descriptor: [Cheese] explode all trees

#4 MeSH descriptor: [Milk] explode all trees

#5 MeSH descriptor: [Milk Proteins] explode all trees

#6 MeSH descriptor: [Cultured Milk Products] explode all trees

#7 MeSH descriptor: [Yogurt] explode all trees

#8 MeSH descriptor: [Lactose] explode all trees

#9 ((milk OR cream*) NEAR/2 (ferment* OR cultur*OR product* OR sour* OR protein*)):ti,ab,kw

#10 {OR #1-#9}

#11 MeSH descriptor: [Skates (Fish)] explode all trees

#12 MeSH descriptor: [Fish Products] explode all trees

#13 MeSH descriptor: [Fish Proteins] explode all trees

#14 MeSH descriptor: [Seafood] explode all trees

#15 MeSH descriptor: [Shellfish] explode all trees

#16 (fish*):ti,ab,kw OR (“sea food”):ti,ab,kw OR ('seafood'):ti,ab,kw OR (surimi):ti,ab,kw

#17 {OR #11-#16}

#18 MeSH descriptor: [Ethanol] explode all trees

#19 MeSH descriptor: [Alcohols] explode all trees

#20 MeSH descriptor: [Caffeine] explode all trees

#21 MeSH descriptor: [Catechin] explode all trees

#22 (ethanol):ti,ab,kw OR (alcohol):ti,ab,kw OR (beer):ti,ab,kw OR (caffeine):ti,ab,kw OR (coffea):ti,ab,kw OR (coffee):ti,ab,kw OR (coffe*):ti,ab,kw OR (tea):ti,ab,kw OR (teas):ti,ab,kw OR ("decaffeinated tea"):ti,ab,kw OR ((tea OR teas) NEAR/3 extract*):ti,ab,kw OR ("camellia sinensis"):ti,ab,kw OR (wine):ti,ab,kw OR (resveratrol):ti,ab,kw OR ('decaffeinated coffee'):ti,ab,kw

#23 (catechin):ti,ab,kw OR ("catechinic acid"):ti,ab,kw OR (cianidanol*):ti,ab,kw OR (ciandiol*):ti,ab,kw OR (cianidol*):ti,ab,kw OR (cyanidanol*):ti,ab,kw

#24 MeSH descriptor: [Coffea] explode all trees

#25 MeSH descriptor: [Coffee] explode all trees

#26 MeSH descriptor: [Tea] explode all trees

#27 MeSH descriptor: [Drinking Behavior] explode all trees

#28 MeSH descriptor: [Wine] explode all trees

#29 resvida:ti,ab,kw OR longivenex:ti,ab,kw OR 'polygonum cuspidatum':ti,ab,kw OR resv:ti,ab,kw OR 'srt 501':ti,ab,kw OR "srt-50":ti,ab,kw OR srt501:ti,ab,kw OR 'trans resveratrol:ti,ab,kw ' OR "red wine":ti,ab,kw OR stilbene:ti,ab,kw OR 'japanese knotweed':ti,ab,kw

#30 MeSH descriptor: [Beer] explode all trees

#31 {OR #18-#30}

#32 (meat*):ti,ab,kw OR ("red meat"):ti,ab,kw OR ('processed meat'):ti,ab,kw OR (pork):ti,ab,kw OR (poultry):ti,ab,kw OR (sausage):ti,ab,kw OR ('animal protein'):ti,ab,kw OR ('animals proteins'):ti,ab,kw OR ('animals protein'):ti,ab,kw OR (bacon*):ti,ab,kw OR ('beef'):ti,ab,kw OR (chicken*):ti,ab,kw OR (lard):ti,ab,kw

#33 MeSH descriptor: [Meat] explode all trees

#34 MeSH descriptor: [Meat Products] explode all trees

#35 MeSH descriptor: [Red Meat] explode all trees

#36 MeSH descriptor: [Chickens] explode all trees

#37 {OR #32-#36}

#38 (bread):ti,ab,kw OR (sorghum):ti,ab,kw OR ('bakery product'):ti,ab,kw OR ('bakery products'):ti,ab,kw OR ('food grain'):ti,ab,kw OR (cereal*):ti,ab,kw OR (corn):ti,ab,kw OR ("corn meals"):ti,ab,kw OR (bulgar):ti,ab,kw OR (bulghar):ti,ab,kw OR (couscous*):ti,ab,kw OR (porridge):ti,ab,kw OR (rice*):ti,ab,kw OR (millet):ti,ab,kw OR (maize*):ti,ab,kw OR (barley*):ti,ab,kw OR (rye*):ti,ab,kw OR (oat*):ti,ab,kw OR (wholegrain*):ti,ab,kw OR (wholemeal*):ti,ab,kw OR (wholewheat*):ti,ab,kw OR ((whole NEAR/3 grain*)):ti,ab,kw OR (whole NEAR/3 wheat):ti,ab,kw OR (whole NEAR/3 food):ti,ab,kw OR (wheat NEAR/3 meat):ti,ab,kw OR (grain*):ti,ab,kw OR (wheat*):ti,ab,kw

#39 MeSH descriptor: [Edible Grain] explode all trees

#40 MeSH descriptor: [Bread] explode all trees

#41 MeSH descriptor: [Sorghum] explode all trees

#42 MeSH descriptor: [Oryza] explode all trees

#43 MeSH descriptor: [Whole Grains] explode all trees

#44 {OR #38-#43}

#45 (plant*):ti,ab,kw OR ('vegetable oil'):ti,ab,kw OR (fruit*):ti,ab,kw OR (anthocyanin):ti,ab,kw OR (anthocyanin*):ti,ab,kw OR (apple*):ti,ab,kw OR (banan*):ti,ab,kw OR (bean*):ti,ab,kw OR (berry):ti,ab,kw

#46 apple*:ti,ab,kw OR banan*:ti,ab,kw OR bean*:ti,ab,kw OR berry:ti,ab,kw OR brassica:ti,ab,kw OR broccoli:ti,ab,kw OR 'brussels sprout':ti,ab,kw OR 'brussels sprouts':ti,ab,kw OR cabbage*:ti,ab,kw OR carrot*:ti,ab,kw OR citrus:ti,ab,kw OR cranberr*:ti,ab,kw OR cucumber*:ti,ab,kw OR 'dry bean':ti,ab,kw OR epigallocatechol:ti,ab,kw OR gallocatechin:ti,ab,kw OR gallocatechol:ti,ab,kw OR 'dietary fiber':ti,ab,kw OR 'dietary fibre':ti,ab,kw OR 'starch':ti,ab,kw OR 'high fiber':ti,ab,kw OR 'high-fiber':ti,ab,kw OR fiber:ti,ab,kw OR 'fruit vegetable':ti,ab,kw

#47 MeSH descriptor: [Plants] explode all trees

#48 MeSH descriptor: [Vegetables] explode all trees

#49 MeSH descriptor: [Plant Oils] explode all trees

#50 MeSH descriptor: [Fruit] explode all trees

#51 MeSH descriptor: [Plants, Medicinal] explode all trees

#52 MeSH descriptor: [Dietary Fiber] explode all trees

#53 MeSH descriptor: [Starch] explode all trees

#54 MeSH descriptor: [Flavonoids] explode all trees

#55 MeSH descriptor: [Isoflavones] explode all trees

#56 MeSH descriptor: [Propyl Gallate] explode all trees

#57 MeSH descriptor: [Spices] explode all trees

#58 MeSH descriptor: [Phytoestrogens] explode all trees

#59 MeSH descriptor: [Polyphenols] explode all trees

#60 MeSH descriptor: [Soy Foods] explode all trees

#61 MeSH descriptor: [Flax] explode all trees

#62 MeSH descriptor: [Agaricales] explode all trees

#63 MeSH descriptor: [Ginkgo biloba] explode all trees

#64 MeSH descriptor: [Isothiocyanates] explode all trees

#65 MeSH descriptor: [Seaweed] explode all trees

#66 MeSH descriptor: [Panax] explode all trees

#67 MeSH descriptor: [Coix] explode all trees

#68 flavonoid:ti,ab,kw OR flavanone*:ti,ab,kw OR flavonoid*:ti,ab,kw OR bioflavonoid*:ti,ab,kw OR flav?none:ti,ab,kw OR flavonone*:ti,ab,kw OR flavone*:ti,ab,kw OR flav?nol:ti,ab,kw or flavonol*:ti,ab,kw OR flavanolignan*:ti,ab,kw OR flavanolignan*:ti,ab,kw OR flav?n:ti,ab,kw OR isoflavone*:ti,ab,kw OR 'epigallocatechin gallate':ti,ab,kw OR 'propyl gallate':ti,ab,kw OR quercetin:ti,ab,kw OR rutin*:ti,ab,kw OR oxerutin*:ti,ab,kw OR anthocyanidin:ti,ab,kw OR catechin:ti,ab,kw OR epicatechin:ti,ab,kw OR naringenin:ti,ab,kw OR hesperetin:ti,ab,kw OR daidzein:ti,ab,kw OR genistein:ti,ab,kw OR hydroxyethylrutoside*:ti,ab,kw OR 'o s hydroxyethyl rutoside':ti,ab,kw OR 'b hydroxyethyl rutoside':ti,ab,kw OR catechin*:ti,ab,kw OR 'procyanidoli oligomers':ti,ab,kw OR 'flavon‐3‐ol*':ti,ab,kw OR 'benzoflavone*':ti,ab,kw OR kaempferol:ti,ab,kw OR myricetin:ti,ab,kw OR apigenin:ti,ab,kw OR luteolin:ti,ab,kw

#69 'fruit juice':ti,ab,kw OR 'vegetable juice':ti,ab,kw OR 'fruit juices':ti,ab,kw OR 'vegetable juices':ti,ab,kw OR juice*:ti,ab,kw OR garlic:ti,ab,kw OR ginger*:ti,ab,kw OR spice*:ti,ab,kw OR tomato*:ti,ab,kw OR lycopene:ti,ab,kw OR onion*:ti,ab,kw OR palmetto:ti,ab,kw OR phytoestrogen*:ti,ab,kw OR 'plant estrogen':ti,ab,kw OR polyphenol*:ti,ab,kw OR punicaceae:ti,ab,kw OR pomegranate*:ti,ab,kw OR potato*:ti,ab,kw OR cucurbita:ti,ab,kw OR pumpkin:ti,ab,kw OR soybean*:ti,ab,kw OR 'soy food':ti,ab,kw OR tofu:ti,ab,kw OR daidzin*:ti,ab,kw OR daidzein*:ti,ab,kw OR genistin*:ti,ab,kw OR genistein*:ti,ab,kw OR glycetin*:ti,ab,kw OR glycetein*:ti,ab,kw OR miso:ti,ab,kw OR spinach:ti,ab,kw OR strawberr*:ti,ab,kw OR 'sweet potato':ti,ab,kw OR 'sweet potatos':ti,ab,kw OR 'ipomoea batatas':ti,ab,kw OR chive*:ti,ab,kw OR flax*:ti,ab,kw OR flaxseed*:ti,ab,kw OR linseed*:ti,ab,kw OR 'mushroom*':ti,ab,kw OR 'fungi':ti,ab,kw OR ginkgo*:ti,ab,kw OR gingko*:ti,ab,kw OR gingkco:ti,ab,kw OR ginko*:ti,ab,kw OR gingho:ti,ab,kw OR gincosan:ti,ab,kw OR bilobalid*:ti,ab,kw OR tebonin:ti,ab,kw OR tebokan:ti,ab,kw OR kaveri*:ti,ab,kw OR tanakan:ti,ab,kw OR tanakene:ti,ab,kw OR rokan:ti,ab,kw OR supergin*:ti,ab,kw OR egb*:ti,ab,kw OR isothiocyanate*:ti,ab,kw OR kelp:ti,ab,kw OR ginseng:ti,ab,kw OR seed*:ti,ab,kw OR leek*:ab,ti

#70 #45 OR #46 OR #47 OR #48 OR #49 OR #50 OR #51 OR #52 OR #53 OR #54 OR #55 OR #56 OR #57 OR #58 OR #59 OR #60 OR #61 OR #62 OR #63 OR #64 OR #65 OR #66 OR #67 OR #68 OR #69

#71 MeSH descriptor: [Fish Oils] explode all trees

#72 MeSH descriptor: [Cod Liver Oil] explode all trees

#73 MeSH descriptor: [Fatty Acids, Omega-3] explode all trees

#74 MeSH descriptor: [Butter] explode all trees

#75 MeSH descriptor: [Dietary Fats] explode all trees

#76 MeSH descriptor: [Fats, Unsaturated] explode all trees

#77 MeSH descriptor: [Fatty Acids] explode all trees

#78 MeSH descriptor: [Fatty Acids, Monounsaturated] explode all trees

#79 MeSH descriptor: [Docosahexaenoic Acids] explode all trees

#80 MeSH descriptor: [Eicosapentaenoic Acid] explode all trees

#81 MeSH descriptor: [Fatty Acids, Volatile] explode all trees

#82 MeSH descriptor: [Margarine] explode all trees

#83 MeSH descriptor: [Oils] explode all trees

#84 MeSH descriptor: [Olea] explode all trees

#85 MeSH descriptor: [Olive Oil] explode all trees

#86 MeSH descriptor: [Oleic Acids] explode all trees

#87 MeSH descriptor: [Thioctic Acid] explode all trees

#88 'fish oil':ti,ab,kw OR 'fish oils':ti,ab,kw OR 'cod liver oil':ti,ab,kw OR 'omega 3 fatty acid':ti,ab,kw OR 'plant oil':ti,ab,kw OR 'plant oils':ti,ab,kw OR butter:ti,ab,kw OR 'fat intake':ti,ab,kw OR 'edible oil':ti,ab,kw OR fat*:ti,ab,kw OR 'fatty acid':ti,ab,kw OR 'monounsaturated fat':ti,ab,kw OR mufa:ti,ab,kw OR 'docosahexaenoic acids':ti,ab,kw OR dha:ti,ab,kw OR pufa:ti,ab,kw OR 'eicosapentanoic acid':ti,ab,kw OR 'eicosapentanoic acids':ti,ab,kw OR epa:ti,ab,kw OR 'volatile fatty acid':ti,ab,kw OR margarine:ti,ab,kw OR oil*:ti,ab,kw OR olive:ti,ab,kw OR olea:ti,ab,kw OR 'oleic acid':ti,ab,kw OR 'perilla seed oil':ti,ab,kw OR 'perilla oil':ti,ab,kw OR 'lipoic acid':ti,ab,kw

#89 #71 OR #72 OR #73 OR #74 OR #75 OR #76 OR #77 OR #78 OR #79 OR #80 OR #81 OR #82 OR #83 OR #84 OR #85 OR #86 OR #87 OR #88

#90 MeSH descriptor: [alpha-Tocopherol] explode all trees

#91 MeSH descriptor: [Tretinoin] explode all trees

#92 MeSH descriptor: [Folic Acid] explode all trees

#93 MeSH descriptor: [Iron] explode all trees

#94 MeSH descriptor: [Magnesium] explode all trees

#95 MeSH descriptor: [Magnesium Sulfate] explode all trees

#96 MeSH descriptor: [Trace Elements] explode all trees

#97 MeSH descriptor: [Dietary Supplements] explode all trees

#98 MeSH descriptor: [Antioxidants] explode all trees

#99 MeSH descriptor: [Vitamins] explode all trees

#100 MeSH descriptor: [Probiotics] explode all trees

#101 MeSH descriptor: [Synbiotics] explode all trees

#102 MeSH descriptor: [Lactobacillus] explode all trees

#103 MeSH descriptor: [Bifidobacterium] explode all trees

#104 MeSH descriptor: [Selenium] explode all trees

#105 MeSH descriptor: [Organoselenium Compounds] explode all trees

#106 MeSH descriptor: [Sodium Selenite] explode all trees

#107 MeSH descriptor: [Vitamin A] explode all trees

#108 MeSH descriptor: [Vitamin B Complex] explode all trees

#109 MeSH descriptor: [Thiamine] explode all trees

#110 MeSH descriptor: [Pyridoxine] explode all trees

#111 MeSH descriptor: [Vitamin B Complex] explode all trees

#112 MeSH descriptor: [Ascorbic Acid] explode all trees

#113 MeSH descriptor: [Vitamin D] explode all trees

#114 MeSH descriptor: [Ergocalciferols] explode all trees

#115 MeSH descriptor: [Cholecalciferol] explode all trees

#116 MeSH descriptor: [Riboflavin] explode all trees

#117 MeSH descriptor: [Molybdenum] explode all trees

#118 MeSH descriptor: [Selenomethionine] explode all trees

#119 MeSH descriptor: [Zinc] explode all trees

#120 MeSH descriptor: [Oxides] explode all trees

#121 MeSH descriptor: [Calcium] explode all trees

#122 MeSH descriptor: [Calcium Carbonate] explode all trees

#123 MeSH descriptor: [Vitamin K] explode all trees

#124 MeSH descriptor: [Vitamin K 1] explode all trees

#125 'alpha tocopherol':ti,ab,kw OR 'tocopherol':ti,ab,kw OR 'vitamin e':ti,ab,kw OR betacarotene*:ti,ab,kw OR ('beta carotene'):ti,ab,kw OR carotene:ti,ab,kw OR carotenoid*:ti,ab,kw OR caroten*:ti,ab,kw OR 'retinoic acid':ti,ab,kw OR 'retinoic acids':ti,ab,kw OR retinoid*:ti,ab,kw OR tretinoin:ti,ab,kw OR 'vitamin a acid':ti,ab,kw OR 'trans-retinoic acid':ti,ab,kw OR 'trans retinoic acid':ti,ab,kw OR 'all-trans-retinoic acid':ti,ab,kw OR 'all trans retinoic acid':ti,ab,kw OR 'beta-all-trans-retinoic acid':ti,ab,kw OR 'beta all trans retinoic acid':ti,ab,kw OR '13 cis retinoic acid':ti,ab,kw OR 'retin a':ti,ab,kw OR 'isotretinoin':ti,ab,kw OR 'folic acid':ti,ab,kw OR 'folate':ti,ab,kw OR 'vitamin m':ti,ab,kw OR 'folacin':ti,ab,kw OR 'pteroylglutamic acid':ti,ab,kw OR iron*:ti,ab,kw OR mgso4:ti,ab,kw OR 'magnesium sulfate':ti,ab,kw OR 'magnesum':ti,ab,kw OR 'trace element':ti,ab,kw OR 'trace elements':ti,ab,kw OR micronutrient*:ti,ab,kw OR 'dietary supplement':ti,ab,kw OR 'mineral supplementation':ti,ab,kw OR 'mineral supplement':ti,ab,kw OR 'mineral supplements':ti,ab,kw OR vitamin*:ti,ab,kw OR 'probiotic agent':ti,ab,kw OR probiotic*:ti,ab,kw OR synbiotic*:ti,ab,kw OR lactobacil*:ti,ab,kw OR 'bifidobacterium':ti,ab,kw OR bifidus:ti,ab,kw OR bifidobacter*:ti,ab,kw OR streptococc*:ti,ab,kw OR lactococc*:ti,ab,kw OR leuconostoc:ti,ab,kw OR pediococc*:ti,ab,kw OR 'beneficial bacteria':ti,ab,kw OR selen*:ti,ab,kw OR 'selenium derivative':ti,ab,kw OR 'methylseleninic acid':ti,ab,kw OR 'methylselenium':ti,ab,kw OR 'sodium selenite':ti,ab,kw OR retinol*:ti,ab,kw OR retinal*:ti,ab,kw OR 'aquasol a':ti,ab,kw OR 'vitamin a':ti,ab,kw OR thiamin*:ti,ab,kw OR niacin*:ti,ab,kw OR cobalamin*:ti,ab,kw OR biotin*:ti,ab,kw OR neurobion*:ti,ab,kw OR 'pantothenic acid':ti,ab,kw OR 'pyridox':ti,ab,kw OR 'benfotiamine':ti,ab,kw OR 'pyridoxine':ti,ab,kw

#126 'vitamin b1':ti,ab,kw OR 'vitamin b 1':ti,ab,kw OR 'vitamin b2':ti,ab,kw OR 'vitamin b 2':ti,ab,kw OR 'vitamin b3':ti,ab,kw OR 'vitamin b 3':ti,ab,kw OR 'vitamin b5':ti,ab,kw OR 'vitamin b 5':ti,ab,kw OR 'vitamin b6':ti,ab,kw OR 'vitamin b 6':ti,ab,kw OR 'vitamin b7':ti,ab,kw OR 'vitamin b 7':ti,ab,kw OR 'vitamin b9':ti,ab,kw OR 'vitamin b 9':ti,ab,kw OR 'vitamin b12':ti,ab,kw OR 'vitamin b 12':ti,ab,kw OR ascorb*:ti,ab,kw OR 'vitamin d':ti,ab,kw OR 'vitamin d2':ti,ab,kw OR 'vitamin d 2':ti,ab,kw OR 'vitamin d3':ti,ab,kw OR 'vitamin d 3':ti,ab,kw OR calciol:ti,ab,kw OR ergocalciferol*:ti,ab,kw OR 'colecalciferol':ti,ab,kw OR 'cholecalciferol':ti,ab,kw OR 'hydroxycholecalciferol':ti,ab,kw OR 'calcitriol':ti,ab,kw OR 'dihydroxyvitamin d3':ti,ab,kw OR 'alphacalcidol':ti,ab,kw OR 'beta tocopherol':ti,ab,kw OR 'gamma tocopherol':ti,ab,kw OR riboflavin*:ti,ab,kw OR 'vitamin g':ti,ab,kw OR 'molybdenum':ti,ab,kw OR 'selenomethionine':ti,ab,kw OR 'zinc':ti,ab,kw OR antioxidant*:ti,ab,kw OR 'anti oxidant*':ti,ab,kw OR calcium:ti,ab,kw OR 'calcium carbonate':ti,ab,kw OR phytomenadione:ti,ab,kw OR phytonadione:ti,ab,kw OR phylloquinone:ti,ab,kw OR phylloquinine:ti,ab,kw OR phyllohydroquinone:ti,ab,kw OR aquamephyton:ti,ab,kw OR menaquinone*:ti,ab,kw OR menadione:ti,ab,kw OR '2 methyl 14 naphthoquinone':ti,ab,kw OR '2 methylnaphthoquinone':ti,ab,kw OR '2 methyl 14 naphthalenedione':ti,ab,kw OR 'acetomenaphthone':ti,ab,kw OR farnoquinone:ti,ab,kw OR menadiol:ti,ab,kw OR menatetrenone:ti,ab,kw OR 'vitamin k':ti,ab,kw OR 'macronutrient*':ti,ab,kw

#127 #90 OR #91 OR #92 OR #93 OR #94 OR #95 OR #96 OR #97 OR #98 OR #99 OR #100 OR #101 OR #102 OR #103 OR #104 OR #105 OR #106 OR #107 OR #108 OR #109 OR #110 OR #111 OR #112 OR #113 OR #114 OR #115 OR #116 OR #117 OR #118 OR #119 OR #120 OR #121 OR #122 OR #123 OR #124 OR #125 OR #126

#128 MeSH descriptor: [Chemoprevention] explode all trees

#129 MeSH descriptor: [Neoplasms] explode all trees

#130 MeSH descriptor: [Leukemia] explode all trees

#131 ('cancer prevention' OR oncoprevention OR chemoprevention OR chemoprevention OR 'cancer chemoprevention' OR 'cancer chemoprevention' OR chemoprophylaxis OR chemoprophylaxis OR chemopreventive OR malignan* OR oncogen* OR cancerogen* OR adeno* OR (tumour*):ti,ab,kw OR carcinom* OR cancer* OR neoplasm* OR oncol* OR leukemia OR leukaemia OR leukemias OR leukaemias OR leucocythaemia OR 'hematologic malignancy' OR 'haematologic malignancy' OR 'hematologic neoplasms' OR absarcoma OR blastoma OR 'myelodysplastic syndrome' OR 'myelodysplastic syndromes' OR 'transient myeloproliferative disorder' OR 'hodgkin disease' OR 'hodgkins disease' OR carcinoma)

#132 {OR #128-#131}

#133 MeSH descriptor: [Molecular Targeted Therapy] explode all trees

#134 MeSH descriptor: [Radiotherapy] explode all trees

#135 MeSH descriptor: [Antineoplastic Agents] explode all trees

#136 'radiation therapy':ti,ab,kw OR radiotherapy:ti,ab,kw OR 'treatment associated cancer':ti,ab,kw OR 'chemotherapy':ti,ab,kw OR pharmacotherapy:ti,ab,kw OR 'cancer immunotherapy':ti,ab,kw OR 'cancer surgery':ti,ab,kw OR 'molecularly targeted therapy':ti,ab,kw

#137 {OR #133-#136}

#138 #132 NOT #137

#139 MeSH descriptor: [Diet Therapy] explode all trees

#140 MeSH descriptor: [Food] explode all trees

#141 MeSH descriptor: [Diet] explode all trees

#142 MeSH descriptor: [Phytotherapy] explode all trees

#143 'diet therapy':ti,ab,kw OR food*:ti,ab,kw OR 'dietary intake':ti,ab,kw OR diet*:ti,ab,kw OR dietetic:ti,ab,kw OR dietary:ti,ab,kw OR beverage*:ti,ab,kw OR herb*:ti,ab,kw

#144 {OR #139-#143}

#145 #10 OR #17 OR #31 OR #37 OR #44 or #70 OR #89 OR #127 OR #144

#146 [**Error**]==>{AND #138, #145}
